# African Swine Fever Virus (ASFV): Immunity and Vaccine Development

**DOI:** 10.3390/vaccines11020199

**Published:** 2023-01-17

**Authors:** Kiramage Chathuranga, Jong-Soo Lee

**Affiliations:** College of Veterinary Medicine, Chungnam National University, Daejeon 34134, Republic of Korea

African swine fever virus (ASFV) is the causative agent of the highly contagious disease African swine fever (ASF), which can result in mortality rates of up to 100% in pigs infected by virulent strains [1]. The ancient sylvatic cycle was identified as the source of the first reported case of ASF in East Africa, causing almost 100% mortality in infected domestic pigs with acute hemorrhagic fever, and infections were subsequently reported in most African countries [2]. The most significant transcontinental occurrence was reported in Georgia in 2007, resulting in a new transmission epoch following subsequent occurrences in Ukraine (2012), Belarus (2013), Lithuania (2014), Romania (2017), and Hungary (2018) [3]. In 2018, a new epidemic was reported in the People’s Republic of China, rapidly taking over most Southeast Asian countries and North Macedonia; Thailand announced its first cases of ASFV infection in 2022 [4,5]. Despite its low zoonotic potential and specific host range, ASF has made a significant socio-economic impact, threatening the global pork industry. According to the World Organization for Animal Health, from January 2020 to January 2022, ASF outbreaks were reported in 35 countries or regions around the world, including 4767 cases (1,043,334 animals lost) in domestic pigs and 18,262 cases (29,970 animals lost) in wild boars [5].

ASFV is a large nucleocytoplasmic double-stranded DNA (dsDNA) virus that belongs to the genus Asfivirus in the family Asfarvirida. ASFV has a linear DNA genome of 170–190 kb, with more than 150 open reading frames (ORFs); however, the specific function of most of the proteins within it remains unclear [6]. Even though ASFV has caused severe damage to the swine industry for a century, a licensed vaccine is not yet available. The critical factor that hinders the development of effective and safe ASF vaccines is the lack of research evidence regarding ASFV pathogenesis and a deep mechanistic understanding of innate immune evasion ASFV strategies. However, recent advances in these innate immune evasion ASFV strategies have uncovered significant knowledge that could play an important role in developing ASFV vaccines. This Special Issue aims to contribute to the current knowledge regarding the immune evasion mechanisms used by ASFV to cause an infection and approaches to vaccine development that are likely to benefit hosts upon subsequent natural infection.

From the perspective of ASFV, it is important to prevent the host innate immune response during the early stage of infection. Since the cGAS–STING signaling cascade plays a critical role as an important cellular signaling pathway that activates type I interferons (IFN) or NF-κB promotors upon DNA virus infection, the ASFV genome encodes several proteins that interfere with cGAS–STING signaling pathway molecules [7]. For instance, ASFV EP364R and C129R interact with 2′3′-cGAMP and exert their nuclease activity to cleave 2′3′-cGAMP, thus downregulating subsequent cGAS-initiated downstream signaling [7]. Moreover, ASFV MGF-505-7R induces the expression of ULK1 autophagy-related proteins to degrade STING [8], and MGF505-11R directly interacts with STING, thereby inducing STING degradation through proteasome, lysosome, and autophagy mechanisms [9]. Recent research shows that the direct interaction of ASFV D117L (p17) with STING interferes with STING and TBK1, IKKϵ interactions [10]. ASFV DP96R targets TBK1 and IκB kinase beta (IKKβ) to negatively regulate IFN-I expression and the induction of NF-κB signaling [11], while ASFV MGF-505-7R interacts with IRF7 and TBK1, degrading IRF7 by the autophagy, cysteine, and proteasome pathways and TBK1 by the proteasome pathway [12]. ASFV I215L recruits RNF138 to inhibit K63-related TBK1 ubiquitination, inhibiting IFN-I production [13]. ASFV MGF360-11L interacts with TBK1 and IRF7, degrading TBK1 and IRF7 through the cysteine, ubiquitin-proteasome, and autophagy pathways [14], while ASFV M1249L suppresses the phosphorylation of TBK1 and interacts with IRF3, consequently inducing IRF3 degradation by the lysosomal pathway [15]. Mentioned above are some of the well-characterized immunosuppressive genes encoded in the ASFV genome. Scientists have successfully defined the mechanisms of action of many immunosuppressive genes, and there are many more to uncover. Therefore, these genes are potential target candidates for the development of live attenuated vaccines.

As already mentioned, the complex nature of the ASFV genome and virus particles has been the main factor that has delayed ASFV vaccine development; therefore, despite the intensive research on different vaccine approaches (Figure 1), promising vaccine candidates are still lacking. Inactivated preparations of the ASF virus (ASFV) “Armenia08” adjuvanted with either Polyge or Emulsigen-D failed to offer protection against ASFV [16]. Since pigs first infected with less virulent strains proved to be protected against the relevant virulent virus [17], there is a possibility that live attenuated vaccines could become the most promising vaccine development option in a short time frame. Live attenuated vaccines have been developed by deleting a single gene or several genes from the ASFV genome. However, protection with LAVs (live attenuated vaccines) directly correlates with the replication level and expressed immunogenic gene number. Therefore, LAVs, with single gene deletion, may provide better protection because of optimum replication and the presence of immunogenic genes. ASFV-G-ΔI177L can protect pigs against the virulent ASFV isolate that is currently circulating in Vietnam, with similar efficacy as that reported against the Georgia strain [18]. Attenuated strains could cause chronic ASF infections in vaccinated pigs, due to the presence of residual virulence and the possibility of reversion to virulence. Therefore, extensive experimental evidence must be obtained before field use. ASFV-G-ΔI177L remains genetically stable and phenotypically attenuated during a five-passage reversion to virulence study in domestic swine; moreover, virus transmission and the shedding have helped to verify ASFV-G-ΔI177L as a safe live attenuated vaccine [19]. Vaccination experiments with ASFV-ΔL7L-L11L-attenuated strains conferred 100% protection against homologous challenge. In addition, recent evidence shows that an LAV developed by deleting EP402R (CD2v) and A238L genes of the Arm/07/CBM/c2 genotype II strain in COS-1 cells could provide 100% protection against the virulent Korean Paju genotype II strain [20]. Nevertheless, more safety and cross-protective ability tests are needed for the commercial vaccine production of LAVs because ASFV consists of duplications, mutations or deletions of certain sequences in the genome of different genotypes, which may lead to changes in virulence [21].

Since it is necessary to discover possible protective antigens, the development of subunit vaccines has trailed behind that of live attenuated vaccines. Subunit vaccines offer safety advantages over attenuated vaccines; however, the majority of the ASFV protective antigens used in the currently available subunit vaccines are not able to provide full protection [22]. By employing the virulent Portuguese ASFV isolate OUR/T88/, previous investigations have shown that CD8+ T cells play a crucial role in protection [23]. As alternate ASF vaccine platforms, DNA vaccines and vector vaccinations have both been investigated in previous studies. Theoretically, the antigens can be produced intracellularly and via MHC I, which is crucial for CD8+ T cell activation, making DNA vaccines and vectors more immunogenic [24,25]. Neither DNA vaccines, vector vaccines, nor prime-boost immunization strategies can provide complete protection. In some investigations, the recombinant proteins of p30/CP204L, p54/E183L, and CD2v/EP402R have been demonstrated to provide partial protection [26]. Specific antigenic epitopes can be determined by software analysis and validation combined with relevant experiments to guide antigen selection in ASF vaccine development. Several research teams have recently conducted large-scale screening of subunit vaccines to discover protective antigens and identify groups of antigens that can effectively block virus replication in pigs [27].

To develop promising live attenuated vaccine candidates on a commercial scale, deleted genes must be carefully fine-tuned to achieve optimal safety and efficacy. Therefore, genes with immune correlations, in addition to protective properties, must be identified. Moreover, it is necessary to identify genes that encode immunogenic proteins that can be altered or deleted to create targets that can distinguish between the diagnosis of infected animals and vaccinated animals (DIVA). Further research is needed to identify the key antigens involved in the induction of protective cells and antibody responses to develop subunit vaccines. This will help to improve the potency of the vaccines and identify genetic formats that can be used to develop a commercial vaccine against ASFV [22]. In summary, this Special Issue presents a current snapshot of the state of ASFV vaccine development and immune evasion mechanisms used by ASFV, which will help to produce a promising, effective, and safe vaccine. Although ASFV vaccine development has proved difficult, there is much to be hopeful about, with a range of promising technological platforms that are currently being applied to resolve this challenge and several candidates that are already under detailed investigation, which may indicate that production can soon be achieved at a commercial level.

## Figures and Tables

**Figure 1:**
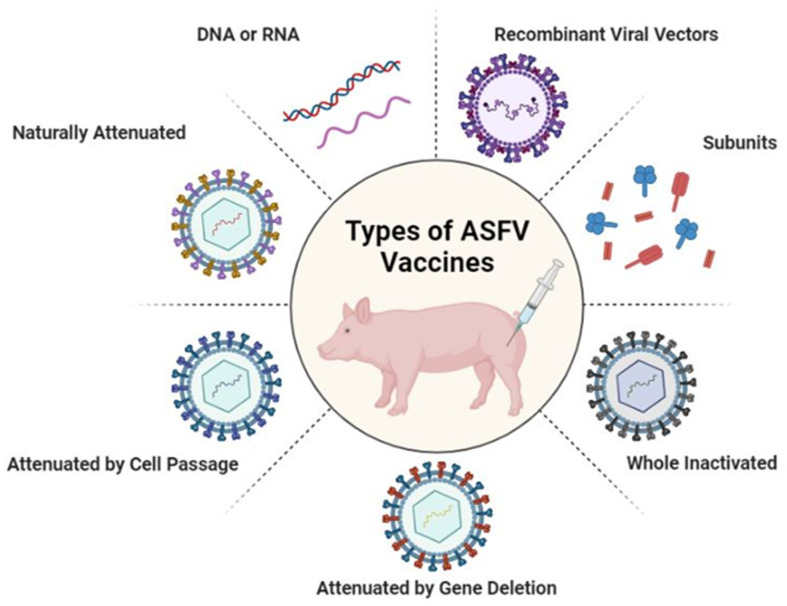
Trends in advances in vaccines against ASFV. Recombinant viral vector vaccines, subunit vaccines, whole inactivated ASF virus vaccines, gene-deleted attenuated vaccines, vaccines attenuated by cell passage, naturally attenuated vaccines and DNA or RNA vaccines. Created with BioRender.com (accessed on 25 December 2022).

## References

[1] Muangkram Y., Sukmak M., Wajjwalku W. (2015). Phylogeographic analysis of African swine fever virus based on the p72 gene sequence. Genet. Mol. Res..

[2] Plowright W., Parker J., Peirce M.A. (1969). African swine fever virus in ticks (Ornithodoros moubata, murray) collected from animal burrows in Tanzania. Nature.

[3] Zhao D., Liu R., Zhang X., Li F., Wang J., Zhang J., Liu X., Wang L., Zhang J., Wu X. (2019). Replication and virulence in pigs of the first African swine fever virus isolated in China. Emerg. Microbes Infect..

[4] Sun E., Huang L., Zhang X., Zhang J., Shen D., Zhang Z., Wang Z., Huo H., Wang W., Huangfu H. (2021). Genotype I African swine fever viruses emerged in domestic pigs in China and caused chronic infection. Emerg. Microbes Infect..

[5] Wang Z., Ai Q., Huang S., Ou Y., Gao Y., Tong T., Fan H. (2022). Immune Escape Mechanism and Vaccine Research Progress of African Swine Fever Virus. Vaccines.

[6] Cackett G., Sýkora M., Werner F. (2020). Transcriptome view of a killer: African swine fever virus. Biochem. Soc. Trans..

[7] Dodantenna N., Ranathunga L., Chathuranga W.A.G., Weerawardhana A., Cha J.W., Subasinghe A., Gamage N., Haluwana D.K., Kim Y., Jheong W. (2022). African Swine Fever Virus EP364R and C129R Target Cyclic GMP-AMP To Inhibit the cGAS-STING Signaling Pathway. J. Virol..

[8] Li D., Yang W., Li L., Li P., Ma Z., Zhang J., Qi X., Ren J., Ru Y., Niu Q. (2021). African Swine Fever Virus MGF-505-7R Negatively Regulates cGAS-STING-Mediated Signaling Pathway. J. Immunol..

[9] Yang K., Huang Q., Wang R., Zeng Y., Cheng M., Xue Y., Shi C., Ye L., Yang W., Jiang Y. (2021). African swine fever virus MGF505-11R inhibits type I interferon production by negatively regulating the cGAS-STING-mediated signaling pathway. Vet. Microbiol..

[10] Zheng W., Xia N., Zhang J., Cao Q., Jiang S., Luo J., Wang H., Chen N., Zhang Q., Meurens F. (2022). African Swine Fever Virus Structural Protein p17 Inhibits cGAS-STING Signaling Pathway Through Interacting With STING. Front. Immunol..

[11] Wang X., Wu J., Wu Y., Chen H., Zhang S., Li J., Xin T., Jia H., Hou S., Jiang Y. (2018). Inhibition of cGAS-STING-TBK1 signaling pathway by DP96R of ASFV China 2018/1. Biochem. Biophys. Res. Commun..

[12] Yang K., Xue Y., Niu T., Li X., Cheng M., Bao M., Zou B., Shi C., Wang J., Yang W. (2022). African swine fever virus MGF505-7R protein interacted with IRF7and TBK1 to inhibit type I interferon production. Virus Res..

[13] Huang L., Xu W., Liu H., Xue M., Liu X., Zhang K., Hu L., Li J., Liu X., Xiang Z. (2022). Correction: African Swine Fever Virus pI215L Negatively Regulates cGAS-STING Signaling Pathway through Recruiting RNF138 to Inhibit K63-Linked Ubiquitination of TBK1. J. Immunol..

[14] Yang K., Xue Y., Niu H., Shi C., Cheng M., Wang J., Zou B., Wang J., Niu T., Bao M. (2022). African swine fever virus MGF360-11L negatively regulates cGAS-STING-mediated inhibition of type I interferon production. Vet. Res..

[15] Cui S., Wang Y., Gao X., Xin T., Wang X., Yu H., Chen S., Jiang Y., Chen Q., Jiang F. (2022). African swine fever virus M1249L protein antagonizes type I interferon production via suppressing phosphorylation of TBK1 and degrading IRF3. Virus Res..

[16] Blome S., Gabriel C., Beer M. (2014). Modern adjuvants do not enhance the efficacy of an inactivated African swine fever virus vaccine preparation. Vaccine.

[17] Detray D.E. (1957). Persistence of viremia and immunity in African swine fever. Am. J. Vet. Res..

[18] Tran X.H., Le T.T.P., Nguyen Q.H., Do T.T., Nguyen V.D., Gay C.G., Borca M.V., Gladue D.P. (2022). African swine fever virus vaccine candidate ASFV-G-ΔI177L efficiently protects European and native pig breeds against circulating Vietnamese field strain. Transbound. Emerg. Dis..

[19] Tran X.H., Phuong L.T.T., Huy N.Q., Thuy D.T., Nguyen V.D., Quang P.H., Ngôn Q.V., Rai A., Gay C.G., Gladue D.P. (2022). Evaluation of the Safety Profile of the ASFV Vaccine Candidate ASFV-G-ΔI177L. Viruses.

[20] Pérez-Núñez D., Sunwoo S.Y., García-Belmonte R., Kim C., Vigara-Astillero G., Riera E., Kim D.-M., Jeong J., Tark D., Ko Y.-S. (2022). Recombinant African Swine Fever Virus Arm/07/CBM/c2 Lacking CD2v and A238L Is Attenuated and Protects Pigs against Virulent Korean Paju Strain. Vaccines.

[21] Bao J., Wang Q., Lin P., Liu C., Li L., Wu X., Chi T., Xu T., Ge S., Liu Y. (2019). Genome comparison of African swine fever virus China/2018/AnhuiXCGQ strain and related European p72 Genotype II strains. Transbound. Emerg. Diseases..

[22] Dixon L.K., Stahl K., Jori F., Vial L., Pfeiffer D.U. (2020). African Swine Fever Epidemiology and Control. Annu. Rev. Anim. Biosci..

[23] Oura C.A.L., Denyer M.S., Takamatsu H., Parkhouse R.M.E. (2005). In vivo depletion of CD8+ T lymphocytes abrogates protective immunity to African swine fever virus. J. Gen. Virol..

[24] Lopera-Madrid J., Osorio J.E., He Y., Xiang Z., Adams L.G., Laughlin R.C., Mwangi W., Subramanya S., Neilan J., Brake D. (2017). Safety and immunogenicity of mammalian cell derived and Modified Vaccinia Ankara vectored African swine fever subunit antigens in swine. Vet. Immunol. Immunopathol..

[25] Sunwoo S.Y., Pérez-Núñez D., Morozov I., Sánchez E.G., Gaudreault N.N., Trujillo J.D., Mur L., Nogal M., Madden D., Urbaniak K. (2019). DNA-Protein Vaccination Strategy Does Not Protect from Challenge with African Swine Fever Virus Armenia 2007 Strain. Vaccines.

[26] Arias M., de la Torre A., Dixon L., Gallardo C., Jori F., Laddomada A., Martins C., Parkhouse R.M., Revilla Y., Rodriguez F.A.J. (2017). Approaches and Perspectives for Development of African Swine Fever Virus Vaccines. Vaccines.

[27] Cadenas-Fernández E., Sánchez-Vizcaíno J.M., Kosowska A., Rivera B., Mayoral-Alegre F., Rodríguez-Bertos A., Yao J., Bray J., Lokhandwala S., Mwangi W. (2020). Adenovirus-vectored African Swine Fever Virus Antigens Cocktail Is Not Protective against Virulent Arm07 Isolate in Eurasian Wild Boar. Pathogens.

